# Effects of intermittent theta burst stimulation on the inflammatory response and cerebral blood flow in promoting neurovascular repair after ischemic stroke

**DOI:** 10.1186/s13041-025-01222-w

**Published:** 2025-06-09

**Authors:** Jingjun Zhang, Siyue Li, Dan Huang, Jiale Fu, Shuying Chen, Na Ren, Pengkun Yang, Di Song, Xiaochen Bai, Hongyu Xie, Gang Liu, Kewei Yu, Shamay S. M. Ng, Junfa Wu, Xiao Xiao, Yi Wu

**Affiliations:** 1 Department of Rehabilitation Medicine, The Sixth People’s Hospital Affiliated to Shanghai Jiao Tong University School of Medicine, Shanghai, China; 2 Department of Rehabilitation Medicine, Huashan Hospital, Fudan University, 12 Wulumuqi Middle Road, Shanghai, 200040 China; 3 Behavioral and Cognitive Neuroscience Center, Institute of Science and Technology for Brain-Inspired Intelligence, Fudan University 2412 East Guanghua Tower, 220 Handan Road, Shanghai, 200433 China; 4 Departments of Laboratory Medicine, Huashan Hospital, Fudan University, Shanghai, China; 5 Department of Rehabilitation Sciences, The Hong Kong Polytechnic University, Hong Kong SAR, China

**Keywords:** Inflammatory response, Ischemia‒reperfusion, ITBS, Neurovascular repair, Stroke

## Abstract

Secondary injuries from ischemia‒reperfusion in stroke, such as edema and hemorrhagic transformation, can significantly impact brain function. This study investigated the effects of intermittent theta burst stimulation (iTBS) on neurological function and cerebral blood flow in a mouse model of ischemia‒reperfusion injury. Laser speckle flowmetry was used to assess changes in cortical blood flow before and after ischemia‒reperfusion. Behavioral assessments were conducted to evaluate motor function recovery. The impact of iTBS on neuronal damage and apoptosis in the peri-infarct area was evaluated via Nissl staining and a TUNEL assay. RNA transcriptome sequencing and immunofluorescence staining were performed to investigate the effects of iTBS on microglial and astrocyte activation and the associated inflammatory response. Our findings demonstrated that iTBS significantly mitigated abnormal perfusion in the infarcted hemisphere, reduced neuronal damage and apoptosis in the peri-infarct area, and enhanced motor function in ischemic mice. Furthermore, iTBS promoted the polarization of microglia and astrocytes toward the anti-inflammatory M2 and A2 phenotypes. Therefore, iTBS provides neurovascular protection by modulating microglial and astrocyte activation and regulating the inflammatory response in the peri-infarct area, thereby improving abnormal cerebral blood flow in both the acute and subacute phases after ischemic brain injury.

## Introduction

Stroke is the second leading cause of mortality globally and is characterized by high incidence, mortality, and disability rates [1]. While numerous neuroprotective therapies have shown promising results in animal models of stroke, their clinical translation has not yielded satisfactory outcomes [2, 3]. Prolonged ischemia followed by reperfusion can disrupt microvascular autoregulation in the brain, leading to ischemia‒reperfusion injury [4, 5]. Despite current guidelines suggesting that all patients who undergo endovascular treatment achieve complete reperfusion, more than one-third of these patients still experience poor outcomes [4]. Thus, timely reperfusion treatment following ischemic injury is critical for attenuating neuronal damage caused by edema and maintaining the integrity of the blood‒brain barrier (BBB) [6]. Therefore, the introduction of the neurovascular unit (NVU) concept has shifted the treatment of ischemic stroke from solely neuroprotective strategies to encompass neurovascular protective therapies [7]. Research indicates that brain functional connectivity is closely linked to dynamic changes in cerebral blood flow, a relationship known as neurovascular coupling (NVC) [8]. Understanding NVC is critical, as ischemic stroke can result in decoupling between neurons and blood vessels [9], which is correlated with poor functional outcomes [10]. Consequently, exploring the mechanisms that underlie NVC may provide insights into improving outcomes for stroke patients. Furthermore, dysfunction in NVC is closely associated with neuroinflammation [11], emphasizing its role in the overall pathophysiology of stroke.

The interaction between microglia and astrocytes, both integral components of the NVU, significantly influences neuroinflammation [11]. Following cerebral ischemia, particularly during the hyperacute phase (1–2 h postreperfusion), M1 proinflammatory microglia become activated and release substantial amounts of proinflammatory cytokines such as IL-1β, IL-6, and TNF-α [12]. Approximately 4 h postreperfusion, these cytokines, especially IL-1β, further activate proinflammatory A1 astrocytes [11], exacerbating neurovascular injury and contributing to brain edema. Studies have shown that astrocytes, as vital structural components of the NVU, are essential targets for promoting functional recovery following ischemic brain injury [13]. These findings highlight the importance of targeting both neurovascular components and inflammatory pathways in therapeutic strategies aimed at enhancing recovery after ischemic events.

Repeated transcranial magnetic stimulation (rTMS) is a safe, noninvasive neuromodulation technique [14, 15] that has been extensively applied in the rehabilitation of clinical stroke patients. By delivering pulsed magnetic fields to the cortical surface, rTMS influences the brain’s metabolism and neuronal electrical activity. Studies have shown that high-frequency stimulation (greater than 5 Hz) can increase neuronal excitability, provide neuroprotection, inhibit inflammatory responses, promote angiogenesis, and regulate local cerebral blood flow [16, 17]. Intermittent theta burst stimulation (iTBS) is a specific form of rTMS characterized by the delivery of bursts of magnetic stimulation at a theta frequency (approximately 5 Hz) in intermittent patterns and has also gained attention in stroke rehabilitation [18–20]. Previous research has indicated [21] [22] that iTBS can modulate the conversion of microglia in the penumbra toward an M2 anti-inflammatory phenotype, thereby reducing the acute inflammatory response associated with brain ischemia and improving neurological and motor functions in ischemic mice. However, while the effects of iTBS on microglial activation have been explored, the long-term effects of iTBS on astrocyte activation, neuronal apoptosis, and vascular protection during ischemia–reperfusion remain largely unexplored. Specifically, the interplay between iTBS-induced changes in microglial inflammatory responses and their subsequent effects on astrocytic function and overall cerebral blood flow is not yet fully understood. Therefore, this study aimed to further investigate the effects of iTBS on the activation of astrocytes, neuronal apoptosis, vascular protection, and long-term effects on cerebral blood flow through the modulation of microglial inflammatory responses in the peri-infarct region.

## Materials and methods

### Animals

This study used SPF-grade male C57BL/6 mice (8 weeks old, 22–24 g) from Shanghai Jihui Experimental Animal Co., Ltd. Upon arrival, the animals were acclimatized for 2 weeks before the start of the experiments. The mice were housed in a temperature-controlled environment (22 ± 1 °C, 50 ± 1% humidity) with a 12-h light/dark cycle and ad libitum access to food and water. The euthanasia of the mice was performed via the intraperitoneal injection of sodium pentobarbital (100 mg/kg–150 mg/kg). This work was performed in accordance with the principles of the Declaration of Helsinki. Approval was granted by the Animal Care and Use Committee of Huashan Hospital, Fudan University (Approval No. 2021 JS Huashan Hospital-128).

### Focal cerebral ischemia/reperfusion

Focal cerebral ischemia/reperfusion was induced by occlusion of the left middle cerebral artery (MCAO), followed by reperfusion. The mice were anesthetized with 1% pentobarbital sodium (100 mg/kg, intraperitoneally) and positioned supine on a heating pad to maintain body temperature (37.0 ± 0.3 °C). A midline neck incision was made to isolate the left common carotid artery (CCA), external carotid artery (ECA), and internal carotid artery (ICA). The CCA and ICA were ligated, and the ECA was incised to insert a silicone-coated 6–0 suture (602156PK; Doccol Corp, USA). The ECA stump was flipped, and the ICA ligation was loosened to advance the suture until resistance indicated focal ischemia onset. After 60 min of ischemia, the suture was removed to allow reperfusion, followed by closure of the neck incision in layers. A topical application of bupivacaine was used for pain relief, and the mice were placed in a warm cage for recovery. In the sham groups, the same protocol was used as in the MCAO/R group, but the middle cerebral artery was not occluded. Instead, a surgical incision is made, and the vessel is manipulated without inducing ischemia.

### Cerebral blood flow (CBF) detection

Cerebral blood flow changes in the mice were measured via a laser speckle flow imaging system (RWD Life Science, Shenzhen, China). After MCAO, the mice were secured in a stereotaxic frame, and a midline incision was made to expose the skull and maintain the hydrogel. The imaging system was activated to locate the target brain region, and parameters, including the image processing mode, spatial filtering constant, frame rate, and recording mode, were set. CBF measurements were taken at baseline, 10 min postischemia, 10 min postreperfusion, and on days 1, 10, and 17 postreperfusion.

### Animal grouping

In the experiment, the number of mice in each experimental group was *n* = 15–25, with the sample size determined on the basis of preliminary experiments, considering Type I error (α), power (1–β), and the modeling success rate. This experiment used the random number table method for grouping (1) the sham group (*n* = 15), (2) the MCAO/R group (*n* = 25), and (3) the MCAO/R + iTBS group (*n* = 25). Throughout the experiment, we made every effort to ensure that all experimental groups were subjected to identical conditions, including proper personnel arrangements, optimal temperature, normal animal physiological rhythms, and the use of blinded detection and data analysis to minimize the influence of both subjective and objective factors.

### rTMS treatment

rTMS was delivered via a device (CCY-II, Wuhan Yirui Medical Equipment, China) with a 64 mm circular coil (peak magnetic output of 3.46 T). Needle electrodes were used to assess motor-evoked potentials (MEPs) in the contralateral gastrocnemius muscle. The resting motor threshold (RMT) was defined as the minimum output required to elicit MEP peak amplitudes exceeding 50 μV in at least 5 of 10 trials, with the average RMT set at 25% of the maximum output (20–30%). The stimulation intensity was set at 100% of the average RMT. The mice in the iTBS group received two daily sessions (10 bursts at 50 Hz, repeated 20 times at 5 Hz intervals) starting 48 h post-MCAO/R injury, with sessions spaced 6 h apart for 7 or 14 days. The sham groups received the same protocol with the coil positioned 15 cm above the head. Prior to iTBS, the mice underwent adaptive grip training to acclimate to the stimulation.

### Behavioral assessments

#### Open field test

The open field test was used to assess exploratory behavior in mice following cerebral ischemia. The experimental setup consisted of a three-dimensional white plastic arena (40 × 40 × 50 cm) and an animal motion tracking system (EthoVision XT15, Noldus, Netherlands). After the system settings were adjusted, the mice were placed at the center of the open field, and their freely moving trajectories were recorded for 5 min. The EthoVision system was used to analyze the total distance traveled and the average speed of each mouse within the arena.

#### Cylinder test

The cylinder test was used to evaluate forelimb usage asymmetry in mice with cerebral ischemia. The apparatus consisted of a transparent cylinder (8 cm diameter, 15 cm height) with mirrors and a high-definition slow-motion camera. The mice were placed in the cylinder, and the frequency with which each forelimb (left, right, or both) touched the walls was recorded over 5 min. The laterality index was calculated via the following formula: Laterality Index = (left + 0.5 × both)/(right + left + both) × 100%.

#### CatWalk gait test

Gait function was assessed via the CatWalk XT gait analysis system (Noldus, Netherlands). A high-speed camera recorded the mouse parameters as they traversed a glass walkway. The mice were acclimatized for 3 days prior to testing. In a quiet, dimly lit room, each mouse was recorded to complete the gait task at least three times. The key metrics included walking duration, standing time, limb swing time, speed, gait cycle, average walking speed, stride length, and gait frequency.

### 2,3,5-Triphenyltetrazolium chloride (TTC) staining

TTC staining was used to assess the infarct area in the mice after cerebral ischemia. The mice were deeply anesthetized with 1% pentobarbital sodium and underwent cardiac perfusion with cold 1X PBS. The brains were extracted and sectioned into 2 mm thick slices via a mouse brain mold along the coronal plane. The slices were incubated in 2% TTC solution (Solarbio, China) at 37 °C in the dark for 20 min, followed by fixation in 4% neutral-buffered formaldehyde (pH 7.4) for 5 min. Unstained areas (white) represent the infarct regions. The infarct area and total hemispheric area were calculated via ImageJ software (National Institutes of Health, USA). The edema index was determined by dividing the left hemisphere area by the right hemisphere area, and the actual infarct area was calculated by dividing the infarct area by the edema index.

### Tissue preparation

Animal samples were subjected to protein blotting, Nissl staining, ELISA for inflammatory factor detection, and immunofluorescence staining. Brain tissue samples were collected via cardiac perfusion with 1 × PBS and subsequently extracted. The tissue was coronally sectioned around the infarct area and quickly frozen in liquid nitrogen before being stored at −80 °C. For Nissl and immunofluorescence staining, brain tissue was perfused with 1 × PBS, fixed in 4% PFA at 4 °C for 24 h, and then dehydrated through 10%, 20%, and 30% sucrose solutions before being sectioned with a cryostat.

### Nissl staining

Nissl staining was used to assess neuronal damage in the peri-infarct region after cerebral ischemia. The frozen sections were dehydrated through 70%, 95%, and 100% ethanol (5 min each) and washed three times with 1 × PBS (5 min per wash). The sections were stained with Nissl staining solution (crystal violet) for 5 min at 37 °C in the dark and rinsed three times with ddH_2_O. The samples were subsequently processed in Nissl differentiation solution for 3 min at room temperature, fixed in anhydrous ethanol for 5 min, air-dried, and mounted with neutral resin.

### TdT-mediated dUTP nick end-labeling (TUNEL)

The TUNEL assay was performed using a kit (Solarbio, China) to detect in situ apoptosis of neurons [22]. The apoptosis index was calculated as the number of TUNEL-positive cells divided by the total number of cells in each field of view. Observations were conducted via an Olympus Fluorview-3000 confocal microscope (Olympus Optical Co., Japan).

### Immunofluorescence staining

Frozen brain tissue Sects. (30 µm thick) were subjected to immunofluorescence staining. The sections were washed three times with 1 × PBS (10 min each), and a hydrophobic barrier was drawn around the edges via an immunohistochemical pen. The samples were incubated with BSA containing 0.3% Triton X-100 (Sigma, Germany) for 20 min, followed by 40 min with additional BSA. The following primary antibodies were applied overnight at 4 °C: anti-NeuN (Abcam, 1:300, UK), anti-NeuN (Proteintech, 1:300, USA), anti-C3d (R&D Systems, 1:200, USA), anti-S100A10 (Proteintech, 1:200, USA), anti-GFAP (Proteintech, 1:300, USA), anti-GFAP (Cell Signaling Technology, 1:300), anti-CD31 (R&D Systems, 1:200, USA), anti-IBA1 (FUJIFILM WAKO, 1:300, Japan), anti-CD86 (Abcam, 1:200, UK), and anti-CD206 (R&D Systems, 1:300, USA) antibodies. After being washed three times with 1 × PBS (5 min each), the sections were incubated with an Alexa Fluor-conjugated secondary antibody (Abcam, 1:1000, UK) for 1.5 h. Finally, the sections were mounted with anti-fade medium containing DAPI (Solarbio, China) for nuclear staining. The samples were observed with an Olympus Fluorview-3000 confocal microscope (Olympus Optical Co., Japan), and ImageJ software (National Institutes of Health, USA) was used for quantitative analysis of the immunofluorescence intensity in each experimental group. The morphological counting analysis in this section involved selecting three distinct fields of view from the peri-infarct region of each brain section for each experimental group of mice and calculating the average value.

### Transcriptome sequencing and analysis

The sequencing technique used was total transcriptome sequencing. The experimental group consisted of 3 samples, and the control group also had 3 samples. The experimental animals were C57BL/6 mice with a stable genotype. The covariate is set at 0.1, the target effect between the experimental group and control group is 1.5, and the false positive rate is 0.05. The statistical power of this experimental design, calculated in RNA Seq Power, is 0.91. The RNA transcriptome sequencing methodology used was described in our previous report [22]. Total RNA was extracted from brain tissue via Trizol reagent (Invitrogen, USA) per the manufacturer's instructions. RNA quantity and purity were assessed with a microultraviolet spectrophotometer, and integrity was verified via an Agilent 2100 Bioanalyzer and agarose gel electrophoresis. The RNA libraries were constructed via the TruSeq RNA Library Prep Kit, and sequencing was performed by LC-Bio Technology Co., Ltd. (Hangzhou, China).

### Statistical analysis

Statistical analysis and data plotting were conducted via GraphPad Prism 9.0 software (GraphPad Software Inc., San Diego, USA). The data are presented as the means ± standard errors of the means (SEMs). The 3σ rule was applied, assuming that the data followed a normal distribution. It is estimated that 99.73% of the data points lie within three standard deviations of the mean, and any values outside this range are considered outliers. No experimental data points were excluded from this study. Differences between groups were compared via one-way analysis of variance (ANOVA), whereas statistical comparisons between two groups were conducted via unpaired t tests. A *p* value of < 0.05 was considered statistically significant.

## Results

### Establishment of the MCAO/R cerebral ischemia mouse model

**Fig. 1:**
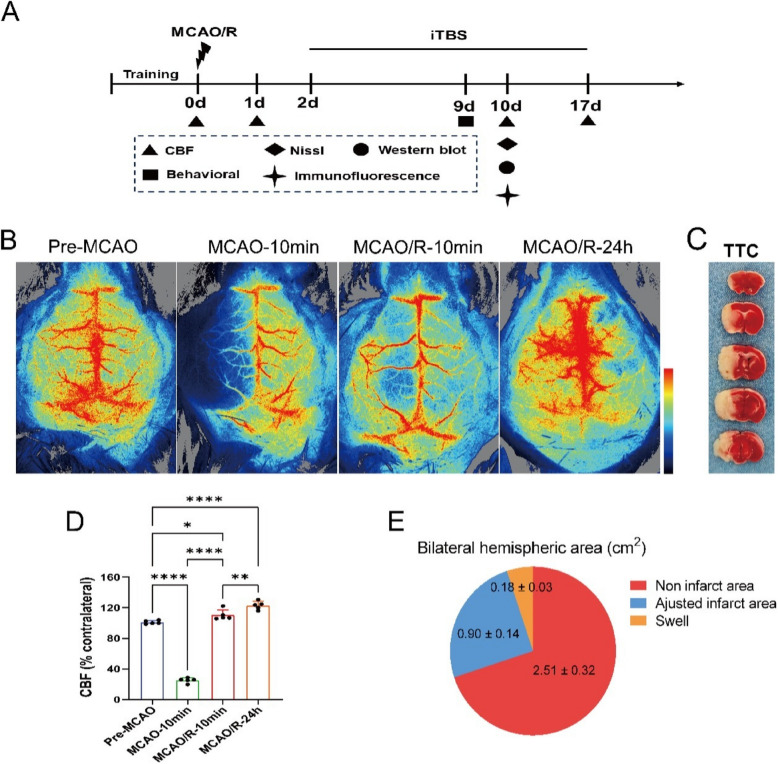
Establishment of the MCAO/R cerebral ischemia mouse model. (**A**) Flowchart of the experiment; (**B**) measurement of cortical blood flow changes in mice at pre-MCAO, 10 min post-MCAO (MCAO-10 min), 10 min postreperfusion (MCAO/R-10 min), and 24 h postreperfusion (MCAO/R-24 h) via a laser speckle flow imaging system; (**C**) TTC staining of brain tissue 48 h post-MCAO/R; (**D**) statistical analysis of bilateral cortical blood flow changes before and after MCAO/R (pre-MCAO vs. MCAO/R-10 min, *P* = 0.03; pre-MCAO vs. MCAO/R-24 h, *P* = 0.0001) (*n* = 5); (**E**) statistical analysis of the infarct area caused by the MCAO/R model (*n* = 5). *P < 0.05, **P < 0.01, ***P < 0.001, ****P < 0.0001

The study flowchart is presented in Fig. 1A, with the onset of MCAO/R marked as Day 0. iTBS began 2 days post-MCAO/R and was administered for either 7 or 14 days. Neurological assessments were performed on Day 9, followed by brain tissue collection on Day 10. Finally, on Day 17, the CBF was re-evaluated to assess the impact of iTBS on blood flow dynamics. Regional blood flow changes in the bilateral hemispheres were assessed via laser speckle flow imaging (Fig. 1B). Successful vascular occlusion was indicated by a ≥ 70% reduction in CBF in the core area of the left middle cerebral artery during the infarction process (Fig. 1D). Blood flow measurements also revealed that the infarct side exhibited hyperperfusion compared with the normal side, with significant differences (Fig. 1D, Pre-MCAO vs. MCAO/R-10 min, *P* = 0.03; Pre-MCAO vs. MCAO/R-24 h, *P* = 0.0001). This hyperperfusion may contribute to increased ischemia‒reperfusion injury. TTC staining (Fig. 1C) was performed to quantify the infarct area, revealing that the infarct size (0.90 ± 0.4 cm^2^) constituted approximately 25% of the total area of the bilateral hemispheres (Fig. 1E).

### iTBS alleviates abnormal hyperperfusion on the infarct side during the acute and subacute phases

**Fig. 2:**
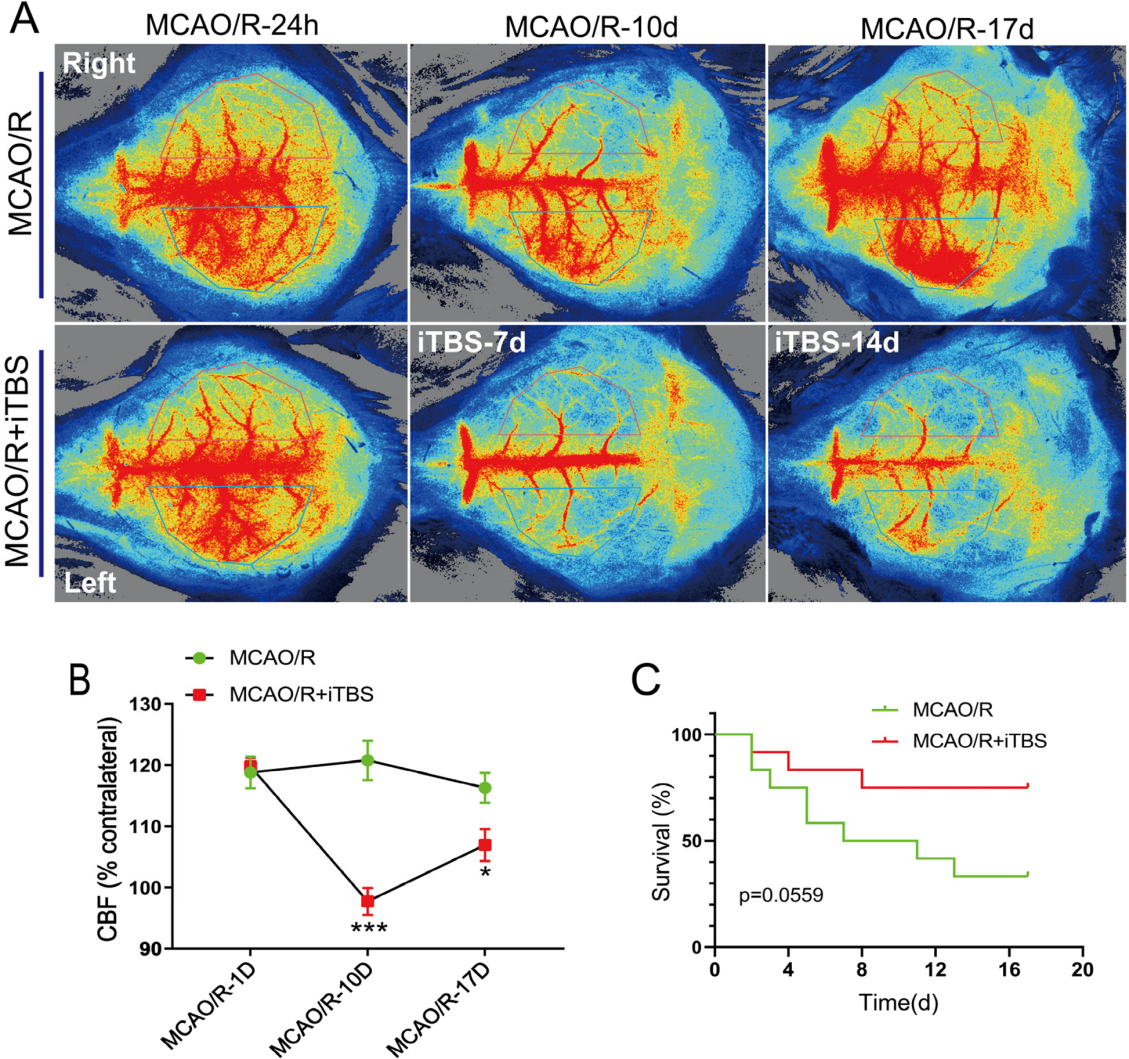
iTBS alleviates abnormal hyperperfusion on the infarct side during the acute and subacute phases. (**A**) Changes in bilateral cerebral blood flow were assessed via laser speckle imaging at 1, 10, and 17 days post-MCAO/R, as well as at 7 and 14 days postiTBS intervention. (**B**) Statistical analysis of bilateral cortical blood flow changes in the MCAO/R group and the MCAO/R + iTBS group (MCAO/R-10D, *P* = 0.0002; MCAO/R-17D, *P* = 0.029) (*n* = 6). (**C**) Survival curve comparison between the MCAO/R group and the MCAO/R + iTBS group (*P* = 0.0559). *P < 0.05, ***P < 0.001

We employed laser speckle imaging (Fig. 2A) to evaluate CBF changes in both the MCAO/R and MCAO/R + iTBS groups on days 10 and 17 postischemia. Statistical analysis (Fig. 2B) revealed that the MCAO/R group sustained elevated CBF levels on the infarct side over the long term. In contrast, hyperperfusion in the core infarct region was significantly lower in the MCAO/R + iTBS group than in the MCAO/R group on days 10 (*P* = 0.0002) and 17 (*P* = 0.029). Additionally, iTBS intervention improved survival rates in the MCAO/R + iTBS group (Fig. 2C, P = 0.0559). These findings suggest that iTBS may alleviate ischemia‒reperfusion injury by modulating excessive blood flow in the infarct area.

### iTBS promotes the recovery of neurological and motor functions in ischemic mice

**Fig. 3:**
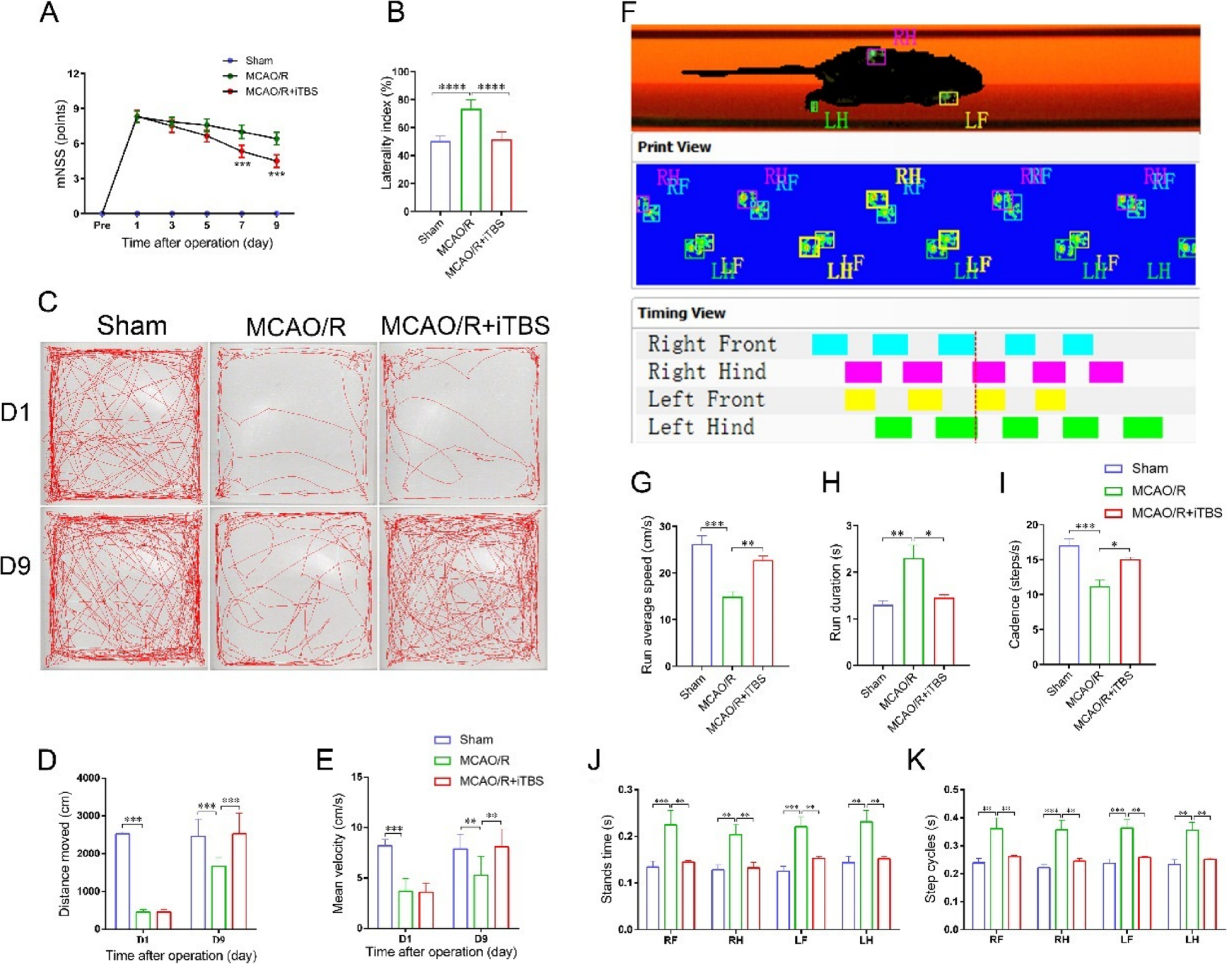
iTBS promotes the recovery of neurological and motor functions in ischemic mice. (**A**) mNSS scores over time for each group; (**B**) Statistical analysis of the paw preference index in the bilateral forelimbs of the mice in the cylinder test; (**C**) Movement trajectories of the mice in the open field test; (**D**-**E**) Statistical analysis of total distance traveled and average movement speed in the open field test; (**F**) Gait analysis trajectory of the mice on the CatWalk glass runway in the CatWalk gait test; (**G**-**K**) Analysis of CatWalk gait parameters, including average walking speed, duration, distance, mean velocity, stand time and speed cycles across groups. *P < 0.05, **P < 0.01, ***P < 0.001. Abbreviations: RF: Right Front; RH: Right hind; LF: Left Front; LH: Left Hind. (The right side of the body is the affected side)

mNSS scoring (Fig. 3A) revealed that, compared with the MCAO/R group, the MCAO/R + iTBS group had significantly lower neurological deficit scores after 7 days (*P* < 0.001). The results of the cylinder test (Fig. 3B) revealed a significant decrease in the ipsilateral bias index in the MCAO/R + iTBS group (51.48 ± 5.48%) compared with the MCAO/R group (73.42 ± 6.55%), suggesting that iTBS enhances neurological recovery and bilateral forelimb coordination. In the open field test (Fig. 3C-3E), the movement distance and speed of the mice in the MCAO/R group were significantly lower than those of the control mice on day 1 postsurgery (*P* < 0.001). After 7 days of iTBS intervention, the MCAO/R + iTBS group presented significant increases in both movement distance (Fig. 3D, *P* < 0.001) and mean velocity (Fig. 3E, *P* < 0.01) compared with the MCAO/R group. Finally, CatWalk gait analysis (Fig. 3F-3K) revealed that iTBS significantly reduced standing time (Fig. 3J, *P* < 0.01) but increased average movement speed (Fig. 3G, *P* < 0.01) and step cadence (Fig. 3I, *P* < 0.05) in the MCAO/R + iTBS group compared with the MCAO/R group.

### iTBS decreases neuronal apoptosis in the peri-infarct area

**Fig. 4:**
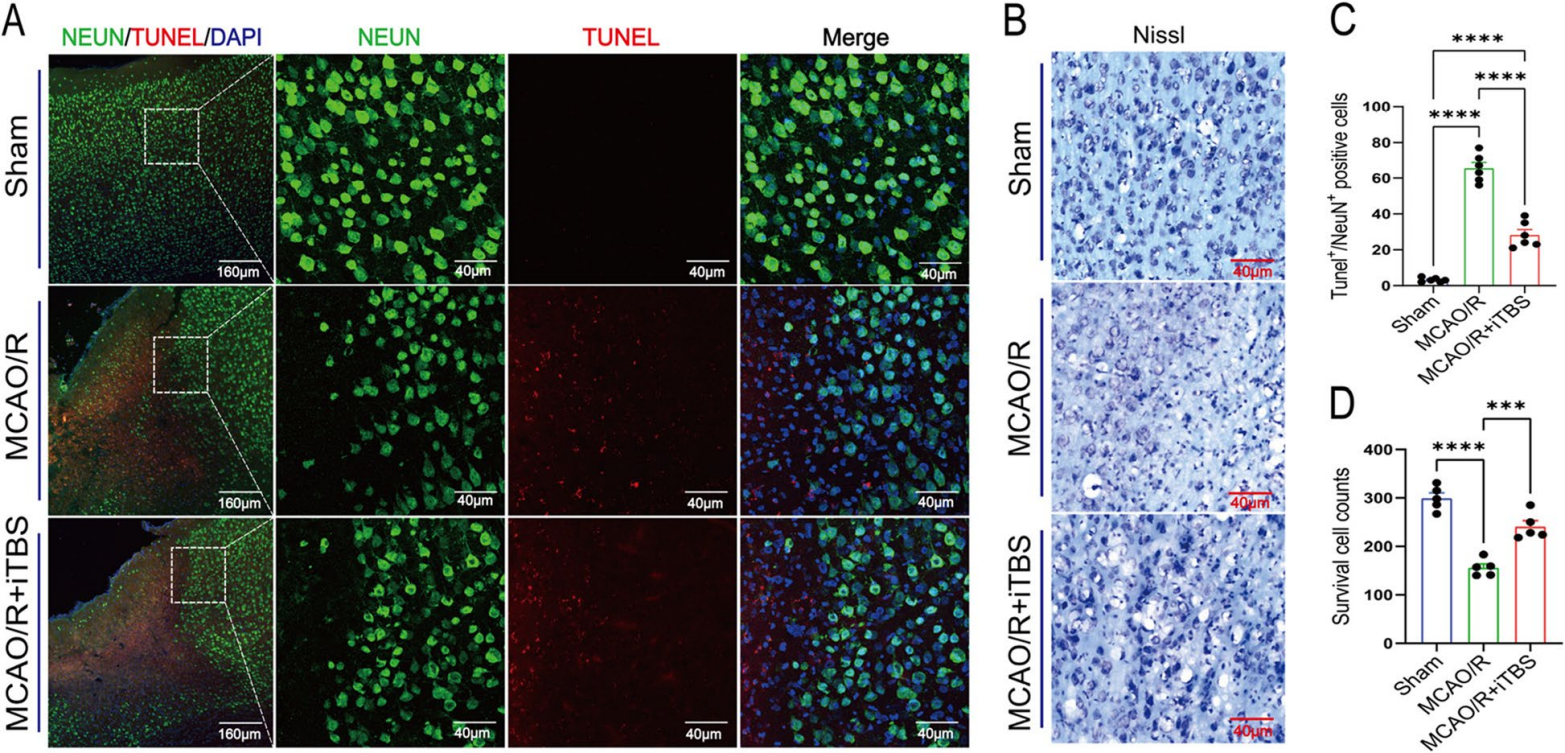
iTBS decreases neuronal apoptosis in the peri-infarct area. (**A**) NeuN/TUNEL costaining was used to detect neuronal apoptosis in the peri-infarct region. (**B**) Nissl staining was used to assess neuronal damage in the peri-infarct area. (**C**-**D**) Quantitative analysis of neuronal apoptosis and survival in the peri-infarct region (MCAO/R group vs. MCAO/R + iTBS group, *P* < 0.001) (scale bars: 40 μm). *P < 0.05, **P < 0.01, ***P < 0.001

TUNEL/NeuN costaining (Fig. 4A) revealed that, compared with MCAO/R alone, MCAO/R + iTBS significantly reduced the number of apoptotic cells and promoted neuronal survival (Fig. 4C, *P* < 0.001). Nissl staining (Fig. 4B) revealed disorganized neuronal arrangements and fewer or no Nissl bodies in the MCAO/R group; in contrast, the MCAO/R + iTBS group exhibited a substantial increase in surviving Nissl bodies (Fig. 4D, *P* = 0.0003).

### iTBS mitigates the inflammatory response in the peri-infarct area

**Fig. 5:**
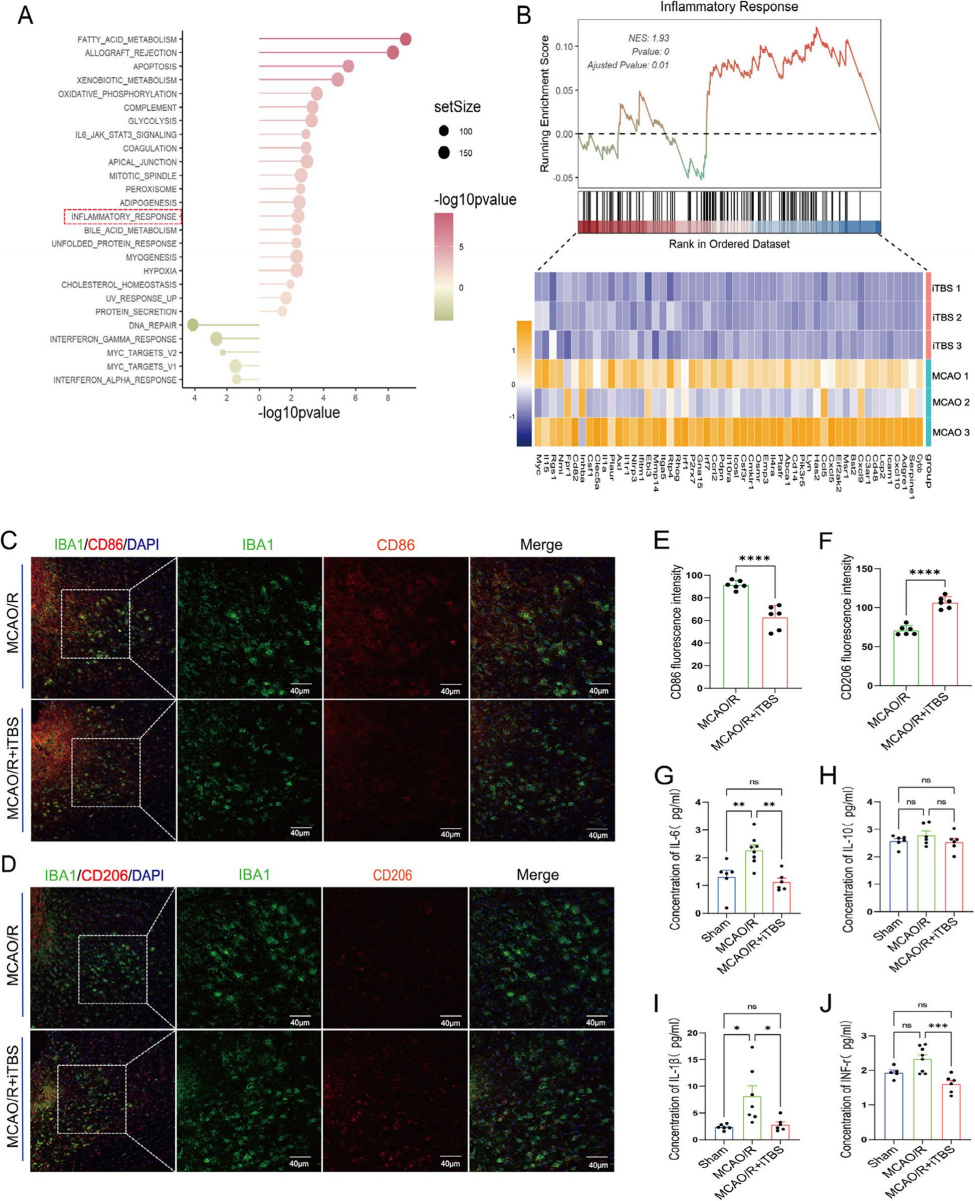
iTBS mitigated the inflammatory response in the peri-infarct area. (**A**-**B**) RNA transcriptome sequencing analysis revealed that iTBS downregulates the expression of multiple genes associated with inflammatory responses. (**C**-**D**) Immunofluorescence staining revealed IBA1 (green) in the peri-infarct area, identified with markers for proinflammatory CD86 (red) and anti-inflammatory CD206 (red) microglia, in the two groups with or without real iTBS. (**E**–**F**) Statistical analysis of the fluorescence intensity of the markers CD86 and CD206. (**G**-**J**) ELISA results showing the expression of the inflammatory cytokines IL-6, IL-10, IL-1β, and IFN-γ in the peri-infarct area. *P < 0.05, **P < 0.01, ***P < 0.001

RNA transcriptome sequencing analysis of the peri-infarct brain tissue from the MCAO/R and MCAO/R + iTBS groups revealed that iTBS plays a role in modulating the acute inflammatory response following cerebral ischemia (Fig. 5A-B). Immunofluorescence staining (Fig. 5C-D) revealed that iTBS significantly increased the expression of the anti-inflammatory marker CD206 (Fig. 5E, t = 9.02, *P* < 0.001) while decreasing the expression of the proinflammatory marker CD86 (Fig. 5F, t = 6.325, *P* < 0.001) in microglia in the MCAO/R + iTBS group. These findings indicate that iTBS facilitates the polarization of microglia toward the M2 anti-inflammatory phenotype. Additionally, ELISA revealed that, compared with the MCAO/R group, the MCAO/R + iTBS group presented significantly lower expression of proinflammatory cytokines, including IL-6 (Fig. 5G, *P* = 0.0015), IL-1β (Fig. 5I, *P* = 0.025), and IFN-γ (Fig. 5J, *P* = 0.0008), in the peri-infarct area. There was no significant change in the expression of the anti-inflammatory cytokine IL-10 (Fig. 5H, *P* > 0.05). These findings suggest that iTBS may effectively modulate microglial polarization and subsequently reduce the inflammatory response in the peri-infarct area.

### iTBS inhibits astrocyte activation in the peri-infarct area, promoting vascular protection

**Fig. 6:**
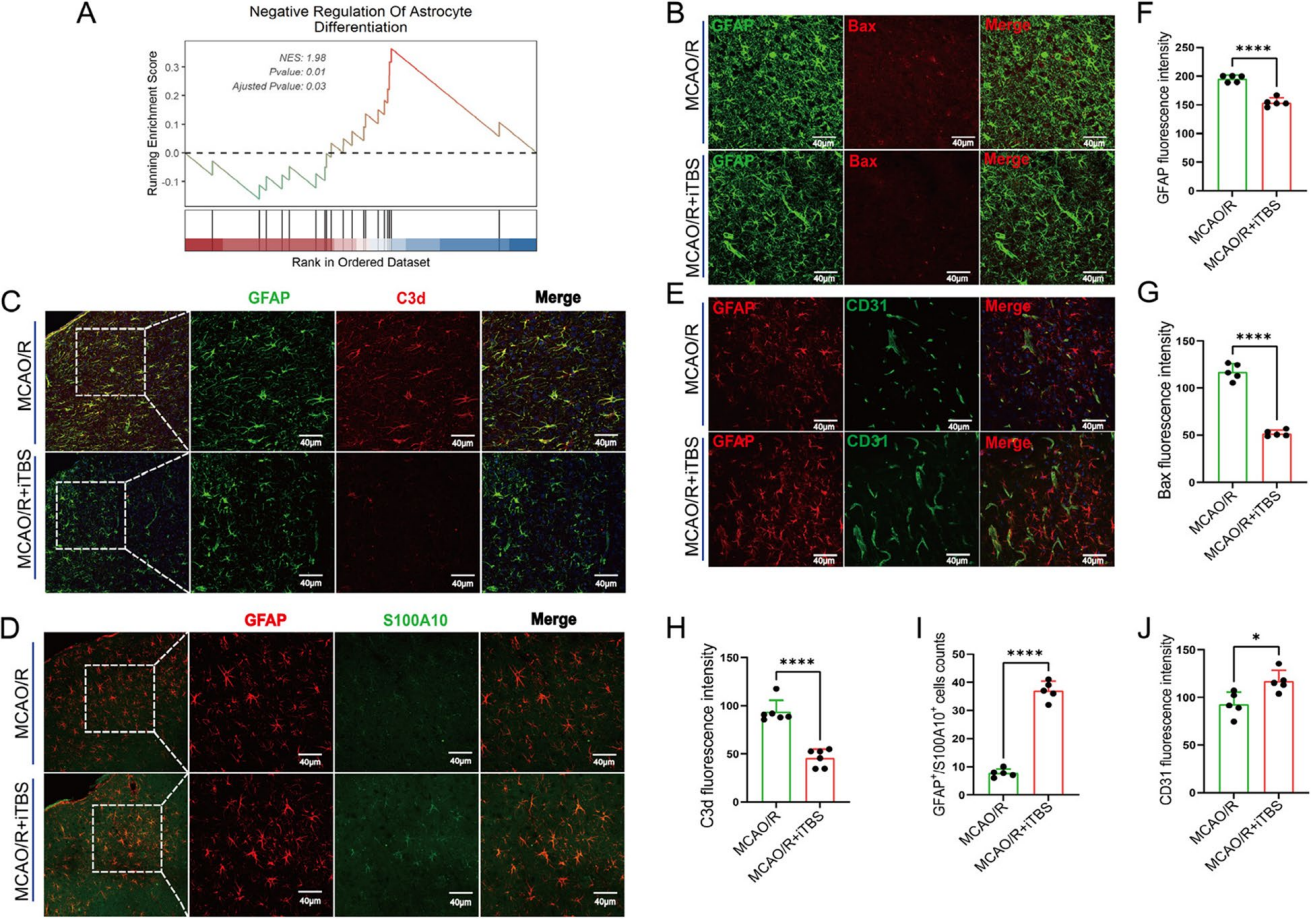
iTBS inhibits astrocyte activation in the peri-infarct area, promoting vascular protection. (**A**) Transcriptomic analysis indicating that iTBS negatively regulates the activation of astrocytes. (**B**) Costaining of the astrocyte marker GFAP (green) with the apoptosis marker Bax (red). (**C**-**D**) Costaining of the astrocyte marker GFAP with the A1-type astrocyte marker C3d (red) and the A2-type astrocyte marker S100A10 (green). (**E**) Costaining of the astrocyte marker GFAP (red) with the vascular marker CD31 (green) (scale bars: 40 μm). (**F**-**J**) Quantitative analysis of the fluorescence intensity of the immunofluorescence markers GFAP, Bax, C3d, S100A10, and CD31 in the peri-infarct area of the MCAO/R group and the MCAO/R + iTBS group. *P < 0.05, **P < 0.01, ***P < 0.001

The RNA transcriptome sequencing results indicated that iTBS negatively regulates astrocyte activation (Fig. 6A). Costaining of the astrocyte marker GFAP with the apoptosis marker BAX (Fig. 6B) revealed that iTBS significantly reduced BAX expression in the peri-infarct area of the MCAO/R + iTBS group compared with that in the MCAO/R group (Fig. 6G, *P* < 0.001) and effectively inhibited aberrant astrocyte activation (Fig. 6F, t = 9.452, *P* < 0.001). Additionally, costaining for GFAP and the A1-type astrocyte marker C3d (Fig. 6C) and the A2-type marker S100A10 (Fig. 6D) revealed that iTBS promoted S100 A10 expression (Fig. 6I, t = 17.68, *P* < 0.001) while suppressing C3d expression (Fig. 6H, t = 7.814, *P* < 0.001) in the MCAO/R + iTBS group. Notably, the number of vascular branch structures enveloped by astrocyte processes was markedly increased. Furthermore, costaining for GFAP and the vascular marker CD31 (Fig. 6E) revealed that iTBS enhanced the coupling between astrocytes and blood vessels, maintaining vascular density and continuity in the MCAO/R + iTBS group compared with the MCAO/R group (Fig. 6J, t = 3.135, *P* = 0.0135). Collectively, these findings suggest that iTBS effectively suppresses the aberrant activation of astrocytes in the peri-infarct area, thereby contributing to vascular protection.

## Discussion

In this study, we investigated the effects of iTBS on microglial and astrocyte activation in the peri-infarct area of a mouse model of ischemic brain injury, with a focus on recovery of the integrity of the NVU. Our findings demonstrated that iTBS significantly enhances neurological and motor functions by reducing neuronal apoptosis, mitigating inflammatory responses, and inhibiting aberrant astrocyte activation. Immunohistochemistry revealed that iTBS effectively polarizes microglia and astrocytes toward anti-inflammatory M2 and A2 phenotypes, respectively, facilitating neurovascular repair and promoting neurovascular coupling, which is essential for maintaining adequate blood flow in response to neuronal activity. Additionally, iTBS mitigated abnormal hyperperfusion in the ischemic hemispheres during both the acute and subacute phases. Collectively, these findings underscore iTBS as a promising therapeutic strategy for mitigating ischemic brain injury and enhancing neuroprotection and recovery.

Reperfusion following ischemia is a critical factor that exacerbates ischemic brain injury. Normally, cerebral perfusion pressure is maintained between 60 and 160 mmHg, with the CBF at approximately 50 ml per 100 g of brain tissue per minute [23]. Deviations from this range can impair the autoregulation of blood flow; consequently, the inability to maintain appropriate cerebral vascular resistance (CVR) may disrupt the BBB and lead to edema [24, 25]. Clinical studies indicate that approximately 40–50% of patients experience hyperemia within three days following an acute stroke [26]. In our study, we observed a significant increase in CBF in the infarcted hemisphere 10 min after ischemia and reperfusion, followed by excessive perfusion after 24 h. TTC staining revealed substantial edema in the infarcted brain tissue (edema index of 0.18 ± 0.03), suggesting that impaired autoregulation occurs after ischemia and reperfusion, contributing to brain edema.

rTMS is a noninvasive neuromodulation technique widely used in clinical settings that is capable of simultaneously modulating cortical neuronal excitability and CBF [14, 27]. Previous studies have shown that rTMS can induce transient changes in blood flow in targeted areas [28]. In this study, we utilized laser speckle flowmetry to assess the long-term effects of iTBS on CBF in mice with cerebral ischemia across both the acute and subacute phases. Our findings revealed that severe ischemic injury resulted in a sustained state of elevated perfusion in the infarcted hemisphere. Following continuous iTBS interventions for 7 and 14 days, we noted significant alleviation of the elevated perfusion status of the infarcted hemisphere, along with increased survival rates. Additionally, neurological and behavioral assessments, along with immunofluorescence results, demonstrated that iTBS significantly improved neurological and motor functions while reducing neuronal apoptosis in the peri-infarct region.

Following ischemic brain injury, the CBF in the core infarct region decreases sharply by more than 90%, resulting in rapid neuronal death. In the peri-infarct area, approximately 35% of the blood supply remains, but many neurons may still undergo apoptosis due to disruption of the NVU and impaired energy metabolism [7, 29]. Therefore, recovery of neurological function after a stroke relies on the integrity of the NVU [30, 31]. Microglia, the primary neuroimmune cells in the brain, are among the first to be activated after ischemic injury, and their activation state significantly influences the overall inflammatory response [32]. Our study revealed that iTBS regulates the inflammatory response in the peri-infarct region by promoting CD206 expression in activated microglia and inhibiting the production of proinflammatory cytokines such as IL-1β, IL-6, and INF-γ.

Studies have shown that microglia begin to activate within 1–2 h following ischemic brain injury [33], whereas astrocytes are activated after 4 h in response to proinflammatory factors released by microglia [34]. Astrocytes constitute approximately half of all neuroglial cells in the brain and serve as crucial links between neurons and blood vessels [35, 36], making them essential for promoting neurological recovery following ischemic injury. Immunofluorescence co-staining of GFAP and BAX showed that iTBS significantly reduced the overall expression levels of GFAP and BAX in the peri-infarct region. These findings suggest that iTBS may alleviate astrocyte overactivation and apoptosis-related signaling in this area. Additionally, morphological observations indicated an increase in astrocytic process alignment around vascular-like structures, although further studies are needed to confirm their functional relevance.

Additionally, colabeling GFAP with the proinflammatory marker C3 d and the anti-inflammatory marker S100 A10 demonstrated that iTBS promoted the conversion of astrocytes to the A2 anti-inflammatory phenotype in the peri-infarct area. Costaining of GFAP with the vascular marker CD31 further revealed that the integrity and continuity of blood vessels in this region were significantly enhanced following iTBS intervention.

However, several questions require further investigation. First, the spatiotemporal characteristics of cerebral infarct outcomes related to varying levels of blood flow perfusion after ischemia and reperfusion need to be clarified. Second, while microglia and astrocytes are activated sequentially after ischemia and reperfusion, further research is needed to determine whether iTBS directly targets astrocytes or indirectly modulates their activity via microglia. Finally, the mechanisms by which iTBS addresses abnormal perfusion and maintains appropriate CVR following ischemia and reperfusion require further exploration.

## Conclusion

In conclusion, our study highlights the therapeutic potential of iTBS in mitigating ischemic brain injury and promoting neurovascular recovery, not only by reducing neuronal apoptosis but also by supporting the integrity of the NVU. By modulating the inflammatory response, polarizing microglia and astrocytes to beneficial phenotypes, and enhancing cerebral blood flow, iTBS has significant neuroprotective effects following ischemia and reperfusion. These findings are crucial for advancing the application of iTBS as a viable treatment option for patients with ischemic stroke.

## Data Availability

No datasets were generated or analysed during the current study.

## Abbreviations

BBB: Blood‒brain barrier
NVU: Neurovascular unit
NVC: Neurovascular coupling
rTMS: Repeated transcranial magnetic stimulation
iTBS: Intermittent theta burst stimulation
CCA: Common carotid artery
ECA: External carotid artery
MCAO: Middle cerebral artery
ICA: Internal carotid artery
CBF: Cerebral blood flow
MEPs: Motor-evoked potentials
RMT: Resting motor threshold
TTC: 2,3,5-Triphenyltetrazolium chloride
TUNEL: TdT-mediated dUTP nick end-labeling
SEMs: Errors of the means
ANOVA: One-way analysis of variance
CVR: Cerebral vascular resistance
